# The impact of post PCNL tube type on blood loss and postoperative pain

**DOI:** 10.12669/pjms.36.3.1558

**Published:** 2020

**Authors:** Salman Jamil, M. Hammad Ather

**Affiliations:** 1 Salman Jamil, Department of Surgery (Urology), Aga Khan University, Karachi, Pakistan; 2 M. Hammad Ather, Department of Surgery (Urology), Aga Khan University, Karachi, Pakistan

**Keywords:** Percutaneous nephrostomy (PCN), Percutaneous Nephrolithotomy (PCNL), Perc, Pain

## Abstract

**Objective:**

To evaluate the impact of nephrostomy tube type on postoperative pain and blood loss following percutaneous nephrolithotomy (PCNL).

**Methods:**

This is a prospective non-randomized study performed at Aga Khan University Hospital from July 2017 to June 2018. In this study we prospectively studied adult patients (16 to 65 years) who underwent unilateral PCNL. Patients who had nephrostomy with balloon (12Fr Foley’s catheter) were compared with patients who had nephrostomy without balloon (12Fr Nelaton™ catheter). STONE Nephrolithometry score was used to assess the stone complexity. Mean pain score at six and 24 hours and mean hemoglobin drop at 24 hours was compared between two groups using independent sample t-test, p-value of <0.05 was considered significant.

**Results:**

Over one year, 198 PCNL were performed out of which 119 were included for analysis. Sixty-six had nephrostomy tube with balloon and 53 had nephrostomy tube without balloon. Mean STONE score (9.66±1.4 vs. 9.64±1.24) and operative time (72.84±28.34 vs. 86.05±32.1 minutes) was comparable. Mean postoperative pain score at 6 hours and 24 hours postoperative was significantly lower in balloon group as compared to without balloon group. Mean Hemoglobin drop was similar in both groups (p=0.60).

**Conclusion:**

The use of nephrostomy tube with balloon after PCNL as this is associated with less pain and comparable hemoglobin drop as compare to nephrostomy tube without balloon.

## INTRODUCTION

PCNL is the standard of care for patients with moderate to large upper tract urinary stones. Despite being minimally invasive it can cause significant pain and sometimes lead to life-threatening complications such as bleeding, sepsis and peri-renal visceral injury.^1^ Multiple factors influence postoperative pain and haemoglobin drop. Most of them e.g. body mass index, stone size and stone location are patient and stone related and cannot be modified.^2^ However there are factors which can be modified to reduce postoperative pain and hemoglobin drop e.g. ultrasound-guided access, use of Amplatz™ or balloon dilatation systems, reducing the operative time, staging the procedure in cases of a large stone burden and placement of a nephrostomy tube.^3^

Nephrostomy tube type is a modifiable factor, which can potentially influence postoperative pain and haemoglobin drop. Su H et al. in 2015^4^ compared the effect of nephrostomy tubes with and without balloon after percutaneous nephrolithotomy. They observed significantly less hemoglobin drop in nephrostomy tube with balloon as compared to those without balloon at 72 hours postoperatively, however noted no difference in postoperative pain. Jiang and colleagues^5^ however noted that the drainage types after PCNL using a nephrostomy tube, a double J stent or an open-ended ureteral catheter were equally safe and efficacious. They also observed that compared to a nephrostomy tube or an open-ended ureteral catheter double J stent adversely affect HRQoL. In a systematic review of recent publications Lee et al.^6^ noted that for hemoglobin changes, total tubeless and finer caliber percutaneous procedure are better than other methods. However, in order to improve hospital stay, total tubeless and tubeless PCNLs with stent may be superior to other procedures. In the current work we have attempted to assess the impact of type of nephrostomy placed post PCNL have influence on postoperative pain and hemoglobin drop.

## METHODS

This is a prospective non-randomized study performed at Aga Khan University Hospital from July 2017 to June 2018. Adult patients 16 to 65 years of age undergoing unilateral PCNL with single tract using 26Fr Amplatz™ sheath for stones in kidney confirmed on non-contrast CT abdomen were included. Patients who previously had open renal surgery on the same side or had a percutaneous nephrostomy already in place or raised serum creatinine (>1.2mg/dL), raised INR (>1.3) or reduced platelet (<100/mm^3^) were excluded.

After institutional review committee approval (Ref: 4649-SUR-ERC-17 dated May 3, 2017), all patients who fulfill the inclusion criteria were included in the study following an informed consent. Consultant surgeon with more than five years of experience performed PCNL. Procedure was done in prone position after placing ureteric catheter in the same general anesthesia using a flexible cystoscope in supine position. Tract was dilated using serial metallic dilators. Stone fragmented with ultrasonic probe using EMS™ lithoclast master. At the end of procedure a 12 Ch., nephrostomy tube, both with or without balloon, was placed and clamped overnight. Patients who had 12 Ch. Foley’s catheter, with balloon inflated (approx. 1-1.5 cc) placed as nephrostomy were included in Group-A. Patients with 12Ch. Nelaton catheter™ placed as nephrostomy were included in Group-B. Choice of nephrostomy was left on operating surgeon’s discretion. Nephrostomy was de-clamped on first post-operative day and subsequently removed on 2-5^th^ postoperative day at the discretion of the admitting surgeon. Post-operative hemoglobin and hematocrit were measured at 24 hours. All patients received paracetamol 1g 6hrly and Tramadol 50mg 8hrly for postoperative pain. Pain was assessed at 6 hours and 24 hours post-operatively.

Data was analyzed through SPSS™ version 21. Mean and standard deviation was calculated for quantitative variables i.e. age, BMI, stone size, density (HU unit) and pain score on VAS. Frequency and percentages were calculated for qualitative variables, i.e. gender, hydronephrosis. Mean postoperative hemoglobin drop was calculated after subtracting 24hr post op hemoglobin from preoperative hemoglobin. of each patient. Mean pain score was calculated at 6 and 24 hrs. Mean postoperative pain and hemoglobin drop of 2 groups were compared using independent sample student t-test. Effect modifiers were controlled through stratification of age, sex, and stone size and STONE score.^7^

## RESULTS

A total of 198 cases had this procedure during the study period out of which 119 were included for final analysis, 66 were in Group-A and 53 in Group-B . Baseline parameters are detailed in Table-I. Both groups were comparable in terms of age, BMI and stone complexity. Complete clearance was achieved in 102 patients and JJ stent was placed in 65 patients. Only four patients required transfusion, none of the patient required angioembolization.

**Table-I:** Baseline parameters.

	Group-A (Foley’s catheter)	Group-B (Nelaton catheter)	p-value
TOTAL (n)	66	53	
Age (years)	44.47+/-13.6	44.94+/-14.5	0.181
Female	18	16	
Male	48	37	
BMI	26.2+/-4.2	26.8+/-3.9	0.418
Stone size	22.27+/-8.03	20.68+/-6.79	0.246
Mean HU	1065.9+/-298.33	1019+/-329.94	0.428
Tract length	85.96+/-23.8	92.0+/-25.25	0.183
Operative time	72.84+/-28.34	86.05+/-32.1	0.021
***Hydronephrosis***			
GRADE 0	9	11	
GRADE 1	12	3	
GRADE 2	22	30	
GRADE 3	18	5	
GRADE 4	5	4	
***No. of calyces involved***			
1	12	5	
2	26	37	
3	16	7	
4	12	4	
STONE Score	9.66+/-1.44	9.64+/-1.24	0.935
Mean pre op Hemoglobin.	13.48+/-2.27	13.64+/-1.90	0.673
Mean 24 hrs. Hemoglobin.	12.40+/-2.18	12.30+/-2.35	0.816
Mean pre op Hct.	41.01+/-5.88	41.59+/-5.22	0.565
Mean 24 hrs. Hct.	37.03+/-7.00	38.23+/-5.63	0.303

There was no significant difference in postoperative Hemoglobin drop between two groups, however mean postoperative pain score at six hours and 24 hours was signifcanlty lower in Group-A (Tables-II and III). Multivariate regression analysis showed that expected mean pain scores at 6 hours is 0.474 units more among those without balloon as compared to those with balloon after adjusting for other covariates. BMI, JJ stenting and Nephrostomy tube were found to be significant independent predictors of pain at 24hours. The expected mean pain scores after 24 hours is 0.389 units more among those without balloon as compared to those with balloon after adjusting for other covariates (Table-IV).

**Table-II:** Comparison of outcome parameters.

	Group-A	Group-B	p-value
Mean PAIN SCORE 6HRS	4.76+/-0.81	5.24+/-0.97	0.004
MEAN PAIN SCORE 24HRS	2.26+/-0.91	2.69+/-1.06	0.020
Mean Hemoglobin drop	1.08+/-0.7	1.14+/-0.69	0.60
Meant Hct drop	3.43+/-2.21	3.41+/-1.97	0.97

**Table-III:** Comparison of outcome parameters.

		Group-A	Group-B	Total	p-value
Complete Clearance	Yes	55	47	102	0.41
	No	11	6	17	
JJ Stenting	Yes	36	29	65	0.98
	No	30	24	54	
No. of Transfusions	Yes	3	1	4	0.26
	No	63	52	114	

**Table-IV:** Multivariate analysis of outcome parameters.

Paint at 6 hours				

	Odds Ratio	Sth. Error	p-value	95.0% Confidence Interval
(Constant)	3.957	0.340	0.000	3.283-4.631
Nephrostomy Tube	0.474	0.165	0.005	0.148-0.801
Gender	0.261	0.181	0.151	-0.097-0.619

*Pain at 24 hours*				

(Constant)	-0.213	0.625	0.734	-1.451-1.025
Nephrostomy Tube	0.389	0.173	0.026	0.047-0.731
BMI	0.071	0.021	0.001	0.029-0.113
JJ Stenting	0.495	0.175	0.005	0.149-0.840

## DISCUSSION

Percutaneous nephrolithotomy (PCNL) despite being minimally invasive can cause significant pain and blood loss.^8^ Post operative pain is the most common concern in immediate post-operative period. There are many ways of reducing postoperative pain. These include local anesthetic infiltration into the tract^9^ reducing the renal pelvic pressure during surgery, paravertebral block (T11-L1), using postoperative opioid analgesia, reducing the size of nephrostomy tube or not placing a nephrostomy tube at all.^10^-^13^

**Fig.1:**
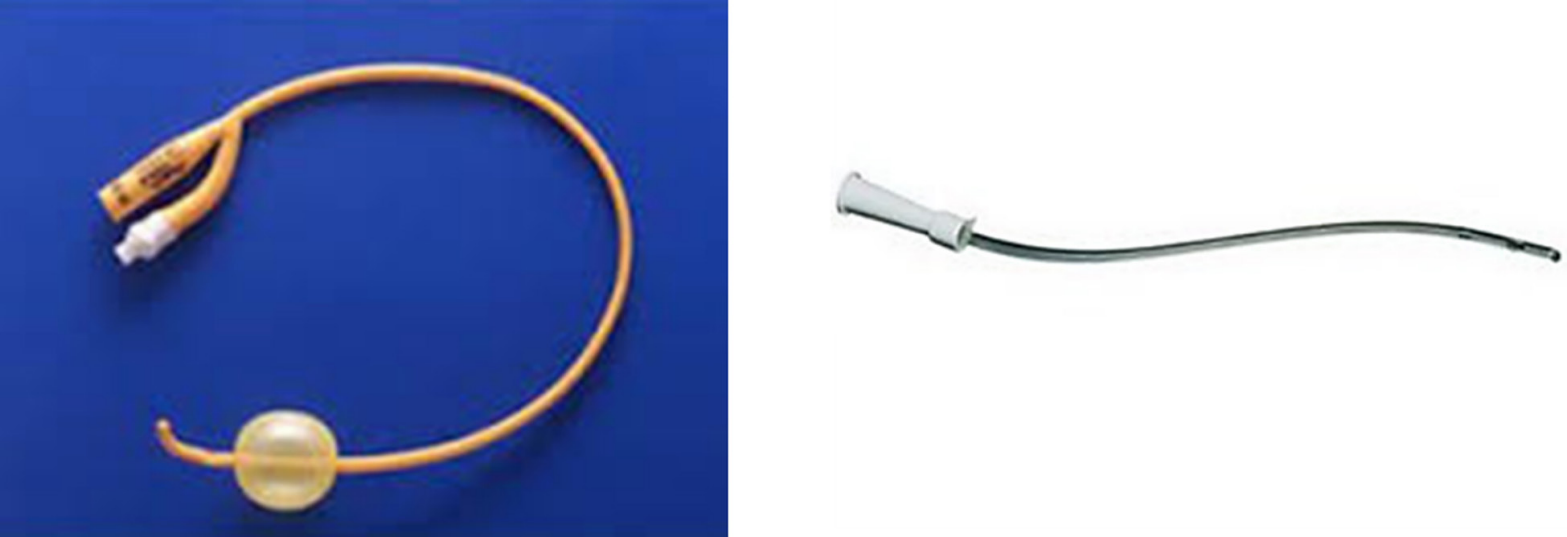
Nephrostomy tube types. 1a: With balloon, 1b: without balloon.

Multiple studies have shown that tubeless PCNL is safe and feasible for select group of patients. However, keeping nephrostomy at end of procedure is desirable in some conditions e.g. residual stone, longer operative time, more than 1 percutaneous access, significant perforation of the collecting system and significant bleeding.^14^ It is well established that decreasing the size of nephrostomy tube can decrease post-operative pain.^15^ However, the effect of nephrostomy tube type on pain is less well studied. We measured pain at six and 24 hours post-operatively and found significantly less pain in balloon nephrostomy group, which may be attributable to softer latex material of balloon tube. In the current work we noted a softer balloon catheter (Foleys 12 Fr.) is much better tolerated compared to a firmer tube (Nelaton™ catherther12 Fr.) Fig.1 a and b.

Postoperative bleeding is another major concern following PCNL. This bleeding is either parenchymal or arterial. Significant delayed bleeding is almost always due to pseudoaneurysms or arteriovenous fistulae. Serious arterial injury requiring angioembolization is around 1% in different studies.^16^ Parenchymal bleeding can be controlled by advancing working sheath during the procedure. However, the tamponade is lost at the end of procedure. This tamponade effect is provided postoperatively by a Foley catheter with a balloon inflated. Arterial bleeding is relatively rare and usually from a tiny arteriole and may occurs during puncture or dilatation of a tract. This type of bleeding can de dealt postoperatively by providing tamponade. Nephrostomy tube with balloon is potentially better for providing this tamponade as shown by Su et al.^4^ they compared nephrostomy tube with balloon and without balloon but found no significant difference in post-operative pain. In their study the primary endpoint was hemoglobin drop and they applied traction over nephrostomy, which can affect pain. Our results showed no differnece in hemoglobin drop between two groups perhaps as we did not keep nephrostomy tube on traction postoperatively that may affect hemoglobin drop.

### Limitations of the study

This was an interventional study however randomization was not done. Nephrostomy tube with balloon was used by single surgeon whereas Nelaton catheter was used by others, this may have contributed to differences in postoperative pain and operative time. Whether to clamp nephrostomy before removal and when to remove ureteric and urethral catheter was left on discretion of operating surgeon.

## CONCLUSION

The present study supports the use of nephrostomy tube with balloon (Foley’s catheter) after PCNL as this is associated with less pain and comparable hemoglobin drop as compare to nephrostomy tube without balloon (Nelaton catheter). Further randomized controlled trails are needed to compare both the tubes.

### Authors’ Contribution:

**SJ and MHA:** Protocol/project development, Data collection or management, Data analysis, Manuscript writing/editing, are responsible for integrity of research.
